# Reversible cardiomyopathy in a patient with Marfan’s syndrome. Case report

**DOI:** 10.47487/apcyccv.v4i3.309

**Published:** 2023-09-30

**Authors:** Adriana E. Viñas-Mendieta, Jesús K. Cárdenas-Gallegos, Roberto Baltodano-Arellano, Fredy Chipa-Ccasani, Gerald C. Lévano-Pachas, Candace Keirns, Nilda Espinola-Zavaleta

**Affiliations:** 1 Hospital Nacional Guillermo Almenara Irigoyen, Lima, Peru. Hospital Nacional Guillermo Almenara Irigoyen Lima Peru; 2 Cardiologist. Hospital Nacional Dos de Mayo, Lima, Peru. Cardiologist. Hospital Nacional Dos de Mayo Lima Peru; 3 Shelby County Health Department, Memphis, TN, USA. Shelby County Health Department Memphis, TN USA; 4 National Institute of Cardiology Ignacio Chavez. ABC Medical Center, P.A.I. Observatorio, Mexico City, Mexico. National Institute of Cardiology Ignacio Chavez. ABC Medical Center, P.A.I. Observatorio Mexico City Mexico

**Keywords:** Marfan’s Syndrome, Arrhythmias, Heart Failure, Case Report, Síndrome de Marfan, Arritmias, Insuficiencia Cardiaca, Caso Clínico

## Abstract

Marfan´s syndrome is a multisystemic, autosomal dominant congenital abnormality of variable penetrance that affects the integrity of connective tissue. In the cardiovascular system, the dysfunction of the physiology of the aortic root and the myocardial fibrosis originates non-ischemic cardiomyopathy independent of valve lesions. Few data have been reported on the prevalence of arrhythmias and its impact on heart function. We present a 21-year-old man with Marfan’s syndrome and heart failure with frequent supraventricular arrhythmias and aortic root dilation. After ablation in the posteroseptal area of the mitral ring and Tirone David Surgery, there was clinical improvement, the left ventricular ejection fraction increased dramatically from 33% to 46%, the left ventricular end-diastolic volume decreased from 90 ml/m^2^ to 77 ml/m^2^ and the NT-proBNP decrease from 1100 pg/mL at 180 pg/mL.

## RESUMEN

El síndrome de Marfan es una anomalía congénita multisistémica, autosómica dominante y de penetrancia variable que afecta a la integridad del tejido conectivo. En el sistema cardiovascular, también se ha descrito la disfunción de la fisiología de la raíz aórtica y la fibrosis miocárdica que origina una miocardiopatía no isquémica independiente de las lesiones valvulares. Se han comunicado pocos datos sobre la prevalencia de arritmias y su repercusión en la función cardiaca. Presentamos el caso de un varón de 21 años con síndrome de Marfan e insuficiencia cardiaca con frecuentes arritmias supraventriculares y dilatación de la raíz aórtica. Después de la ablación en la zona posteroseptal del anillo mitral y la cirugía de Tirone David, hubo mejoría clínica, la fracción de eyección ventricular izquierda aumentó espectacularmente del 33 al 46%, el volumen telediastólico ventricular izquierdo disminuyó de 90 a 77 mL/m* y el NT-proBNP disminuyó de 1100 a 180 pg/mL.

## Introduction

Marfan syndrome (MSx) is a multisystemic, autosomal dominant congenital abnormality of variable penetrance that affects the integrity of connective tissue. The incidence is 1:5000 with no predilection for gender, ethnicity, or geographic distribution. It presents as a new mutation in 25% of cases, and in 90%of cases it is caused by mutations in the Fibrillin-1 gene (FBN1) located on chromosome 15Q21.1 ^1^. FBN1 is a 350 KDa glycoprotein secreted by fibroblasts and incorporated as insoluble microfibrils that allow the deposition of elastin, a principal component of the extracellular matrix of such structures as skin, lungs, kidneys, blood vessels, cartilage, tendons, muscles, cornea and ciliary zonule ^2^. The diagnosis of MSx is based on the revised criteria of Ghent, which focus on cardiovascular manifestations ^3^. In the cardiovascular system, the primary manifestation is dilatation of the aortic root and proximal ascending aorta, leading to aortic dissection, the main cause of death ^1^. Myocardial fibrosis that originates non-ischemic cardiomyopathy independent of valve lesions has also been described. Few data have been reported on the prevalence of arrhythmias ^4^.

## Case report

**Figure 1:**
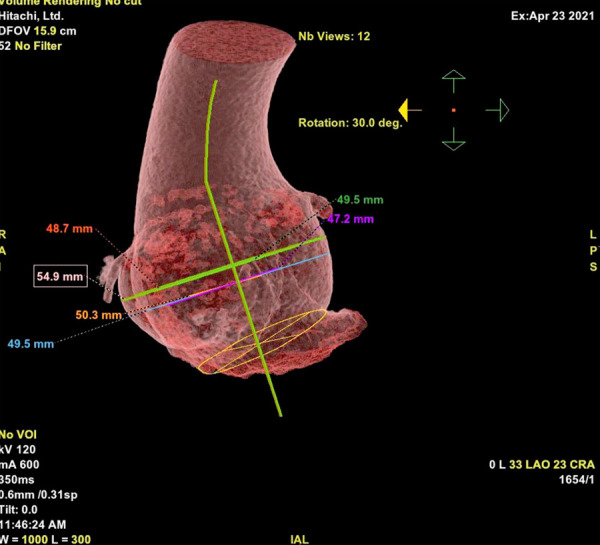
Three-dimensional tomographic reconstruction of the dilated aortic root with a maximum diameter of 54.9 mm.

The patient was a 21-year-old man diagnosed with Marfan´s syndrome (MSx) at the age of 10. He was treated with 5 mg of nevibolol daily. The patient reported progressive shortness of breath and palpitations over 4 years that deteriorated to NYHA functional class III. Transtoracic echocardiography showed left ventricular systolic dysfunction with a left ventricular ejection fraction (LVEF) of 33%, left ventricular end-diastolic volume of 90 ml/m^2^, eccentric hypertrophy, type I diastolic dysfunction with E/A ratio of 0.57 and E/e’ in 14, right ventricle of normal dimensions and function (TAPSE 24 mm, S’ wave 11 cm/sec). There was left atrial dilatation with a volume of 40 ml/m^2^, aneurysmal dilatation of the ascending aorta (52 mm), no significant valvulopathies and no signs of pulmonary hypertension; it is very important to mention that the patient had a suboptimal sonographic window due to his thoracic anatomy. The tomographic study revealed a dilated aortic root **(**Figure 1**)**. 

**Figure 2:**
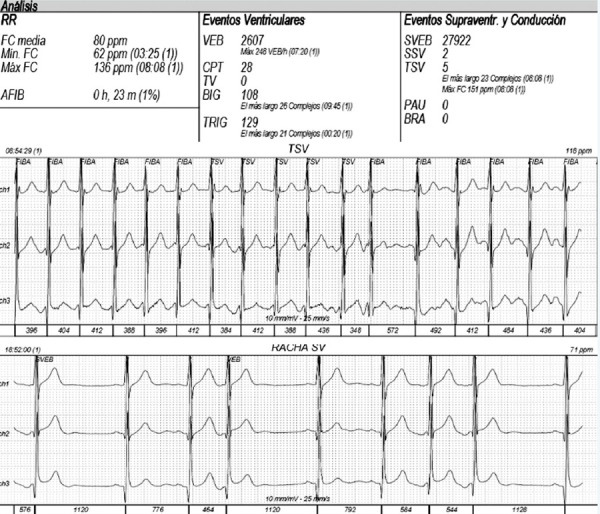
24-hour electrocardiographic monitoring pre-ablation reveals frequent supraventricular escape beats (27%), as well as 5 episodes of atrial tachycardia, the longest including 23 beats with a heart rate of 151 bpm.

**Figure 3:**
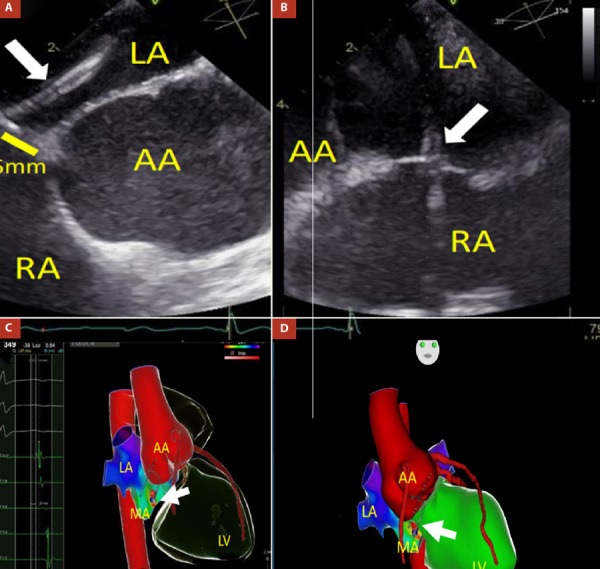
(A and B) Transseptal puncture guided by transesophageal echocardiography: transseptal sheath and needle (white arrow) are directed from RA to LA and located 5 mm from AA. Three-dimensional Electroanatomic Mapping fusioned with computed tomography shows a catheter ablation positioned in the poster-septal region of the mitral annulus. C) Right anterior oblique (RAO) view: Local activation time (LAT) was -30ms from the atrial coronary sinus electrogram, as determined from both bipolar and unipolar electrograms. D) Anteroposterior (AP) view: Radiofrequency catheter ablation (white arrow) in the posteroseptal region of the mitral annulus, near to dilated aortic root.

LA: left atrium; AA: aortic annulus; MA: mitral annulus; LV: left ventricle; RA: right atrium.

Twenty-four-hour electrocardiographic monitoring revealed frequent supraventricular premature atrial complexes (PACs) around 27% of total beats, and five episodes of atrial tachycardia at 150 bpm **(**Figure 2**)**. The NT-proBNP was 1100 pg/mL. Unfortunately, cardiac magnetic resonance imaging was not performed because this method was not available in the hospital. As no cause of cardiomyopathy apart from MSx and supraventricular arrhythmias was found, an electrophysiological study and ablation of the arrhythmogenic focus was pursued. Ablation was performed with radiofrequency at the level of the posteroseptal region of the mitral ring **(**Figure 3**)**. Three months later, the heart team decided to replace the aorta with a dacron tube (Tirone David surgery) without complications. 

After the aforementioned procedures, the patient reported considerable clinical improvement and functional capacity NYHA I. The 24-hour electrocardiographic monitoring showed a decrease in the frequency of PACs to 2.3%. Echocardiographic evaluation 8 months after the ablation showed improvement of the LVEF to 46%, left ventricular end-diastolic volume of 77 ml/m^2^, normal diastolic function with E/A ratio of 1.5, E/e´ in 11 and mild dilatation of the LA (diameter 40 mm/m^2^). The NT-proBNP control was 180 pg/mL. 

## Discussion

Arrhythmia-induced cardiomyopathy is a ventricular condition caused by a persistent arrhythmia involving irregular, asynchronous, and/or rapid contractions that produce hemodynamic, electrical, and structural changes and neurohormonal activation ^5^. Recently, atrial fibrillation, re-entry tachycardias, atrial tachycardia, and frequent supraventricular escape beats have been reported to cause cardiomyopathy ^6^^-^^8^.

Fatima Ezzedine et al. assessed the prevalence of arrhythmias in 213 patients with MSx. They concluded that atrial arrhythmias occurred in 16% of them, and 95% of these were atrial fibrillation, 3% atrial flutter, and 3% paroxysmal supraventricular tachycardia. Ventricular tachycardias were detected in 5% ^4^. Douglas Mah et al. studied the prevalence of arrhythmias in 274 children and young adults between 6 months and 25 years of age. In this group, 7% presented ventricular and 5% supraventricular arrhythmias. They concluded that although premature ventricular escape beats (PVCs) and PACs in MSx had low incidence, the PVCs were associated with a higher left ventricular end-diastole volume and PACs with a larger diameter of the sinotubular junction ^9^.

In a study of 846 patients, Sampath Gunda et al. found no association between PACs and cardiomyopathy and suggested that irregularity and post-extrasystolic potential produced by PACs and PVCs played no pathophysiological role in cardiomyopathy. However, these findings cannot be generalized given that the patients had short-term follow-ups and only 62 patients had > 5% PACs ^10^. At present, there are few reports of frequent PACs cases developing atrial and ventricular cardiomyopathy that improves with successful ablation ^10^.

In conclusion, we present the case of a patient with MSx in whom two important components that contributed to heart failure were identified: the first was the high frequency of supraventricular extrasystoles with episodes of atrial tachycardia, and the second was the dysfunction of the physiology and dynamics of the aortic root due to its dilatation. Ablation of the arrhythmogenic focus and Tirone David surgery were performed; both procedures contributed to the clinical and quality of life improvement of the patient, and could also be demonstrated by the echocardiographic and laboratory control.
